# FLIM-MAP: Gene Context Based Identification of Functional Modules in Bacterial Metabolic Pathways

**DOI:** 10.3389/fmicb.2018.02183

**Published:** 2018-09-18

**Authors:** Vineet Bhatt, Anwesha Mohapatra, Swadha Anand, Bhusan K. Kuntal, Sharmila S. Mande

**Affiliations:** ^1^Bio-Sciences R&D Division, TCS Research, Tata Consultancy Services, Pune, India; ^2^Academy of Scientific and Innovative Research (AcSIR), CSIR-National Chemical Laboratory, Pune, India

**Keywords:** gene context, functional potential, bacteria, metabolic pathway, annotation, genome

## Abstract

Prediction of functional potential of bacteria can only be ascertained by the accurate annotation of its metabolic pathways. Homology based methods decipher metabolic gene content but ignore the fact that homologs of same protein can function in different pathways. Therefore, mere presence of all constituent genes in an organism is not sufficient to indicate a pathway. Contextual occurrence of genes belonging to a pathway on the bacterial genome can hence be exploited for an accurate estimation of functional potential of a bacterium. In this communication, we present a novel annotation resource to accurately identify pathway presence by using gene context. Our tool FLIM-MAP (Functionally Important Modules in bacterial Metabolic Pathways) predicts biologically relevant functional units called ‘GCMs’ (Gene Context based Modules) from a given metabolic reaction network. We benchmark the accuracy of our tool on amino acids and carbohydrate metabolism pathways.

## Introduction

**FIGURE 1:**
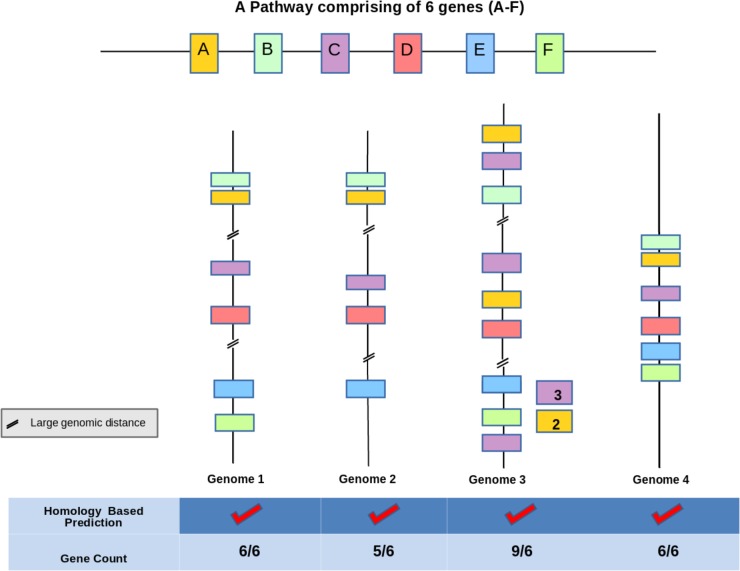
Demonstration of the limitations of sequence homology based pathway prediction algorithms. In this figure the hypothetical pathway ‘A–F’ comprises of six genes representing different candidate bacterial genomes Genome 1, 2, 3, 4. The figure illustrates four cases of gene arrangement where Genomes 1 and 2 do not follow the operon model as they have all the constituent six genes but dispersed at distant locations on the genome, Genome 3 has multiple homologs of the constituent genes dispersed on the genome. These dispersed homologs may function in pathways other than A–F. Genome 4 consists of all the six genes lying within context and represents the actual pathway. Homology based methods might inaccurately predict pathway presence in all the four cases. The actual presence of pathway is observed only in Genome 4 which can be correctly captured by gene context based method.

Functional potential of an organism can be attributed to the set of metabolic pathways which are comprised of a series of reactions catalyzed by enzymes encoded in its genome. Thus, proper annotations of genes and their assignments to correct pathways are crucial for deciphering functional capability of a bacterium. Majority of the pipelines (KEGG, BRENDA, MetaCyc, etc.) utilized for predicting enzymes functional in a pathway from a bacterial genome sequence, rely only on sequence homology and fail to identify proteins with divergent sequences (for e.g., pseudouridine synthase) (Hoang and Ferre-D’Amare, 2004). In addition to missing out remote homologs, sequence homology based methods (KEGG, BRENDA, MetaCyc etc.) might lead to inaccurate prediction of a pathway on the genome in several scenarios. This has been illustrated in **Figure 1** which depicts a demonstrative example of a hypothetical pathway on a bacterial genome comprising six genes (A–F). While Genome 4 in **Figure 1** depicts the correct annotation of the pathway, the other three Genomes (1–3) represent mis-annotations due to homology based methods. An overestimation of functions might occur in cases where homologs of some enzymes constituting a candidate pathway participate in other pathways within the same bacterium (for e.g., promiscuous enzymes like dehydrogenases etc.) (**Figure 1** Genome 1). Thus, it is important to distinguish cognate homologs of each enzyme functioning in a pathway from their other homologs on a bacterial genome in order to accurately decipher functional potential of an organism. Further, accounting for mere presence of homologous enzymes often leads to an inaccurate annotation of a pathway in cases where all constituent enzymes have homologs that function in multiple pathways (e.g., fatty acid degradation, sugar metabolism, butanoate metabolism etc.) (**Figure 1** Genomes 2 and 3). In these genomes, presence of all homologs which might be separately functioning in multiple pathways might be misinterpreted as indicative of pathway presence. In other words, homology based methods may successfully provide a phenotypic function of a protein but might prove inaccurate in identifying its molecular function (Prakash and Taylor, 2012). Thus, pathway prediction tools (e.g., KEGG, MetaCyc, and BRENDA) (Kanehisa and Goto, 2000; Karp et al., 2000; Schomburg et al., 2004) which mostly rely on these homology based methods for gene annotation might lead to an overestimation of functional potential as discussed above.

**FIGURE 2:**
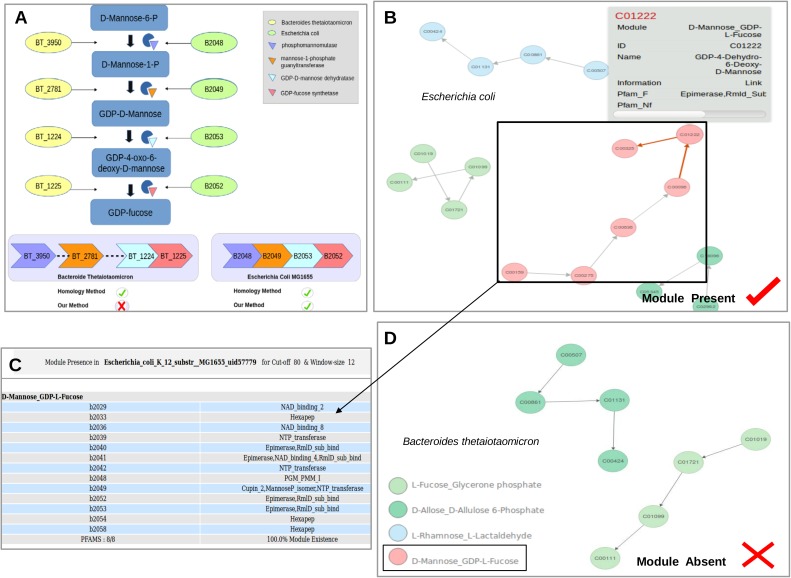
**(A)** Representation of the evolutionarily conserved *Man* gene cluster and its corresponding enzymes involved in the conversion of Mannose to GDP-Fucose in *Escherichia coli* and *Bacteroides thetaiotaomicron*. **(B)** Reorganization of the genes within the Pathway into functionally relevant modules (GCMs) by considering gene context information. The GCM ‘D-Mannose to GDP-L-Fucose’ was present in *E. coli* and its corresponding pfams for the chosen cut-off and window size are highlighted in **(C)**. **(D)** Absence of the module in *B. thetaiotaomicron.*

Reports have indicated that physical as well as functional interactions between proteins are directly correlated with the spatial proximity of their genes on the genome. It is now well established that bacterial metabolic pathways are encoded by co-regulated genes lying in juxtaposition (evolutionarily conserved gene clusters and/or operons) on the genome for improved efficiency (Overbeek et al., 1999; Tamames, 2001; Ballouz et al., 2010). These findings indicate that this clustering of genes forming a candidate pathway on the genome can be utilized to address the above-mentioned issues with pathway annotation and lead to improvement in prediction accuracy (**Figure 1** Genome 4) (Huynen et al., 2000; Gabaldón and Huynen, 2004). Earlier studies have utilized flanking genes information not only for functional annotations of orphan enzymes, but also for identifying pathways to which they belong (Huynen and Snel, 2000). Furthermore, use of genomic context information can help in identifying the correct homologs of enzymes belonging to a pathway (ignoring the other copies which lie on far away locations on the genome) in a bacterium. For example, the homologs for conversion of GDP-mannose to fucose are present in *Bacteroides thetaiotaomicron* as well as *Escherichia coli.* However, the *man* gene cluster required for this conversion is only present in *E. coli* (**Figure 2A**). Therefore, utilization of gene context information can help in correct pathway annotations leaving out spurious results in genomes which merely contain homologs of genes constituting a pathway.

The above mentioned repositories (KEGG, BRENDA, MetaCyc etc.) depict the pathways using a network view, with nodes as compounds (either reactants or products) and edges as reactions. Although the graphical network displays the unified entirety of the pathway, it may not be a biologically relevant representation. For example, in addition to connections involving sets of reactants and products which represent the pathway under consideration, the depiction of pathway in such repositories include other reactions that are not part of this main pathway (**Supplementary Figure S1**). For example, prediction of methane producing capability of a bacterium would require annotation of the gene cluster which is involved in methanogenesis on the genome (Klein et al., 1988; Reeve et al., 1997). However, the network representation corresponding to Methane metabolism in KEGG (Pathway ID: 00680) includes not only this gene cluster, but also other constitutive pathways like formate metabolism, glycolysis, pentose phosphate and serine metabolism. It should be noted that the capability of an organism to metabolize methane will only depend on presence of methane metabolic gene clusters on its genome while involvement of above mentioned additional pathways is expected to cause ambiguity in pathway assignment. Thus, in order to predict the capability of a bacterium to produce a specific final product (called ‘target’) using series of enzymes from an initial reactant (called ‘source’), only the enzymes involved in that pathway should be considered. Therefore it is important to identify biologically relevant modules and prioritize enzymes involved in various pathways.

Automated community detection algorithms as well as manual curation have been utilized earlier in order to identify and annotate the functional units from bacterial genomes (Girvan and Newman, 2002; Newman and Girvan, 2004). However, since automated community detection algorithms rely primarily on network parameters like edge connectivity while defining modules within a pathway, they do not necessarily predict biologically relevant modules. In order to address this, the edge connectivity information needs to be augmented with details of pathways utilized by bacteria to convert a given reactant to a specific product. This information can be obtained using gene context based identification of gene clusters forming these pathways and aid in identifying biologically relevant modules from KEGG pathways.

The present tool, called FLIM-MAP uses a novel method for identifying functional units within a metabolic pathway by utilizing prioritization of gene context information in addition to sequence similarity as well as edge connectivity information (**Figures 2B–D**). Considering the ubiquity of gene clusters in bacterial metabolic pathways, FLIM-MAP assigns appropriate relevance to each cluster (i.e., sub-network within the larger framework of a pathway) by first ensuring connections between compounds (reactants and products) within a metabolic network and further checking the gene neighborhood of the catalyzing enzymes. A valid functional unit is that which not only forms appropriate edge connections (reactions) in a network but also has genes which occur in context within the genome.

## Results and Discussion

A metabolic pathway (as depicted in repositories like KEGG) can be segregated into several functional units, each responsible for converting a specific reactant into a product catalyzed by a set of enzyme(s). These functional units are usually encoded by a set of co-regulated genes which occur in juxtaposed arrangement in the genome. In the current work, a methodology has been devised which allows to augment the modules derived by network parameters with gene context information. The FLIM-MAP algorithm identifies functional units, termed as ‘Gene Context based Modules’ (GCMs), which represent biologically relevant entities with defined metabolic activities within a metabolic pathway. The identification of these functional units requires accounting for edge connections within a metabolic network as well as genomic organization of the constituent genes, making it imperative to create a knowledgebase comprising this information.

### Creation of Initial Reference Modules (IRMs)

Initial Reference Modules (IRMs) were created (details in section “Materials and Methods”) using information on edge connectivity between compounds (set of reactants and products) in KEGG repository corresponding to 11 carbohydrates and 13 amino acid metabolism KEGG pathways (**Supplementary Table S1**). The constitutively expressed pathways like Glycolysis, Pentose Phosphate pathway and Citric acid cycle were not included as most of their enzymes do not occur as clusters on the genome. Similarly, Glycine, Serine, and Threonine metabolism pathways were also excluded from the analysis. Community detection algorithm (described in section “Materials and Methods”) resulted in identification of 99 and 186 (total of 285) IRMs in carbohydrate metabolism and amino acid metabolism pathways, respectively.

As discussed in the Section “Introduction,” community detection algorithms which utilize only the information on edge connectivity in a KEGG pathway network may not necessarily represent biologically relevant functional units. The actual experimentally established source-target relationships (i.e., starting reactant to final product) which exist in bacteria can be obtained by augmenting the edge connectivity in pathways with information of genomic locations of constituent genes. Therefore, the juxtaposed occurrence of co-regulated genes on the genome can be utilized to obtain functional units/modules which correctly represent various functions within a bacterium. The outcomes can be further validated using literature mining for each of these source and target. Details of the created IRM along with validation are described below.

### Creation of Refined Modules After Inclusion of Gene Neighborhood

#### Creation of Genome-Pfam Map (DB1)

A genome-Pfam map, termed as ‘DB1,’ was created based on the HMM profiling of 2745 completely sequenced bacterial genomes. This database comprised of the genes in each organism arranged according to their genomic locations and their corresponding domain (Pfams) annotations. This map therefore acts as a master catalog for querying the specific genes while performing module refinement. The created map has been incorporated into the backend database of the web interface called ‘FLIM-MAP’ for faster and efficient querying.

#### Creation of ‘Gene Context Based Modules’ (DB2)

The algorithm for refinement of ‘Initial Reference Modules (IRM)’ on the basis of genomic distances between constituent genes (described in section “Materials and Methods”) has been applied on the 2745 bacterial genomes and benchmarked on all carbohydrate (11) and amino acid (13) KEGG pathways. Initially the algorithm redefined 82 (out of 186 IRMs) amino acid and 93 (out of 99 IRMs) carbohydrate ‘Intermediate modules’ (IM) after reassigning the genes in the IRMs on the basis of genomic locations (details in section “Materials and Methods”). In other words, these IMs corresponded to sub-pathways obtained from clustering of homologs of the constituent genes in each of the KEGG pathway on the basis of genomic locations on the bacterial genomes. Since the IMs were defined using locations of genes present in IRMs, an IM might be formed by one IRM, merging of some IRMs or splitting an IRM into multiple IMs. The Pfam domain information corresponding to genes within each of IMs was also cataloged. Although, these IMs comprised of juxtaposed genes which were expected to represent a biologically functional unit, they were further validated using information in literature regarding experimental characterization of these sub-pathways. This database included only those sub-pathways (IMs) for which experimental details could be verified from literature. The literature based validation involved a combination of automated search in public literature engines using ‘6Q method’ as well as manual curation (described in section “Materials and Methods”) and the obtained results are discussed below.

The modules obtained after filtering IRMs based on gene context to yield IMs as well as verification in published literature were considered as biologically relevant functional sub-pathways within a KEGG pathway and termed as ‘Gene Context based Modules’ (GCMs). The information for each of the GCMs was cataloged as a database (DB2) along with features such as compounds involved, their order of occurrence and the key domains involved in carrying out enzymatic reactions. A total of 54 carbohydrate and 51 amino acid biologically validated GCMs (total of 105 GCMs) were picked from the initial 285 IRMs (**Supplementary Table S2**).

#### Illustrating Utility of GCMs in Obtaining Biologically Relevant Modules

##### Case study 1: mannose to GDP-fucose conversion-fructose and mannose metabolism pathways (KEGG map00051)

The community detection algorithm divided the Fructose and Mannose metabolism pathway in KEGG (map00051) into 7 modules on the basis of edge connectivity. Studies have established that bacteria utilize evolutionarily conserved *man* gene cluster comprising four genes for conversion of Mannose to GDP-Fucose and therefore, the entire reaction set for this conversion should form part of a single module (Andrianopoulos et al., 1998; Jensen and Reeves, 2001). On the contrary, the community detection algorithm divided the pathway into modules such that two genes were found to be present in one module while the other two were in the second module (**Supplementary Figure S2**). Thus, these edge connectivity based communities do not represent the actual biologically relevant functional modules. Module definition using gene context in addition to edge connectivity, as implemented in the created GCMs in this study, was able to correctly put all these genes in one module (**Supplementary Figure S3**). Further, since these modules were defined after literature verification pertaining to bacterial pathways, eukaryotic sub-pathways within KEGG pathways could also be removed. For example, mannan production is specific to eukaryotes and is absent in bacteria. This demonstrates the advantage of utilizing gene context information in identifying biologically relevant modules in bacterial systems. Additionally, *man* gene cluster (gmd,wca manC,manB) comprises of all genes with promiscuous domains and therefore multiple homologs might exist within a bacterial genome. For example, the homologs of this pathway occur in *E. coli* (B2048, B2049, B2053, and B2052) as well as *B. thetaiotaomicron* (BT3950, BT2781, BT1224, and BT1225). The *man* gene cluster was experimentally characterized in *E. coli* and the conversion pathway is absent in *B. thetaiotaomicron. B. thetaiotaomicron* only contains dispersed homologs of constituent enzymes on the genome. In such scenarios, tools like KEGG, BRENDA, and MetaCyc which determine gene function on basis of sequence homology assign and map all the enzymes to this pathway in both these organisms. This would lead to false prediction of the pathway presence in *B. thetaiotaomicron*. Utilization of gene context information can help in obtaining correct pathway annotations (*E. coli*), leaving out spurious results in genomes which merely contain homologs of genes (*B. thetaiotaomicron*).

##### Case study 2: phenylacetate metabolism-phenylalanine metabolism pathway (KEGG map 00360)

The example of phenylacetate metabolism in bacteria also provides an illustration of importance of GCMs in defining functional units within a pathway. Post the community detection algorithm, the Phenylalanine metabolism pathway was distributed into six modules based on edge connectivities. Bacteria metabolize Phenylalanine, an essential amino acid, using phenylacetate as starting intermediate. It has been shown that Phenylacetate metabolism is brought about by an evolutionarily conserved gene cluster (*paa* gene cluster) (Fernández et al., 2006) which was characterized in several bacterial species including *E. coli* and *Pseudomonas putida*. This gene cluster contain 14 genes organized in two divergent catabolic operons, *paa*ABCDEFGHIJK and *paa*Z, and a regulatory operon, *paa*XY. It was observed that the community detection algorithm divided phenylacetate metabolism sub-pathway (comprising above-mentioned 14 genes) into three clusters which did not represent the actual occurrence of pathway in bacteria correctly. The definition of gene context based modules (GCM) which accounts for gene location and edge connectivities can overcome these discrepancies by clubbing all the 14 genes with their respective reactions in the pathway into a single module (GCM Phenylacetate _Succinyl-CoA Metabolism). In this case also, the genes constituting *paa* gene cluster contain multiple homologs in bacteria which might lead to misannotations. Identification of GCMs allows to handle these false positives generated due to homology based annotation as well. Furthermore, it is to be noted that homologs of proteins like paaF (enoyl-CoA hydratase [EC:4.2.1.17]) and paaH (3-hydroxyadipyl-CoA dehydrogenase [1.1.1.157]) form part of other metabolic pathways like Butanoate metabolism and Fatty acid metabolism. Therefore, the two genes (b1395 and b1393, respectively, in *E. coli* K12 MG1655) are also included as part of the above mentioned pathways in homology based methods (KEGG, MetaCyc etc.). KEGG maps b1393 and b1395 to Butanoate metabolism pathway in *E. coli* and shows presence of gene repertoire for conversion of Pyruvate to Crotonyl-CoA. It is known that *E. coli* lacks the ability to produce butanoate using this pathway thereby illustrating that homology based methods (KEGG, MetaCyc) annotate the pathway incorrectly in such cases. On the contrary, since the genes (b1393 and b1395) would only lie in within ‘*paa’* gene cluster, FLIM-MAP would lead to an appropriate assignment of these genes exclusively to the Phenylacetate to Succinyl-CoA Metabolism where they actually function.

#### Literature Mining Using ‘6Q-Strategy’

The 175 IMs defined using the described algorithm for clustering and reassignment of genes was further refined using manual intervention to remove false positives. These false positives occurred in cases where an IRM contained Pfams which are very commonly found in bacteria and therefore their juxtaposed occurrence on the genome might be a chance event and not actually correspond to a pathway. The elimination of these false positives involved identifying the Pfams which were more specific to a module and could help to distinguish these false positive occurrences from actual presence of a module on a genome (for e.g., Removal of the IM – Ascorbate to L-Xylonate/L-Lyxonate Metabolism from list of IMs for Carbohydrate Metabolism and addition of the IM, 2-Oxoisovalerate to 2-Isopropyl-3-oxosuccinate Synthesis to the intermediate module list of amino acids). Some modules were also redefined to get relevant and pathway specific clusters, for instance, metabolism of Homocitrate to Oxoadipate. This yielded 148 final IMs which were further used as input for validation using ‘6Q-Strategy’ (described in section “Materials and Methods”). It should be noted that IMs removed manually in previous step as false positives were also subjected to literature mining in order to further confirm that they did not represent any functional module.

Each output file contained a list of Pubmed IDs along with their associated reference organisms. The results showed that 6Q approach provided literature validation for 127 modules out of the 148 used as query (e.g., Modules such as L-Proline to *trans*-3-Hydroxy-L-proline metabolism, 2-Oxoadipate to 2-Oxoadipic_acid_Glutaryl-CoA Conversion and CDP-glucose to CDP-tyvelose Metabolism were removed from the list of 148 Intermediate Modules-IMs after literature mining and manual curation) (**Supplementary Table S3**). Further, there were modules where reference organisms were eukaryotes and not bacteria. These eight modules were removed after it was confirmed by manual search also that they belonged to eukaryotic pathways and contained no relevance in bacterial systems (for e.g., Lyxonate biosynthesis or Tartrate metabolism to Hydroxypyruvate, Thyroxine biosynthesis, Eumelanin biosynthesis etc.). Additionally, it should be noted that query structure of 6Q approach includes matches with only reactant string, only product string, reactant and product string combined as well as Reactant string, Product String and the keyword ‘bacteria’ combined (see section “Materials and Methods”). Therefore, considering pubmed IDs present in four out of six query searches as consensus pubmed IDs would even include the Pubmed IDs which only match these four criteria and obtain no publication corresponding to keywords like ‘gene cluster’ and ‘operon.’ In such cases, although the conversion between initial reactant and final product may be captured in a publication but the genes responsible might not occur in gene context (operon or gene cluster) on the genome. As an example, conversion of D-Ribulose-5-phosphate to CDP-ribitol has been discussed in the publication with Pubmed ID 11305920 (Zolli et al., 2001) with reference to organism *Haemophilus influenza* as predicted by 6Q approach with Pubmed ID. Although the conversion of reactant to product has experimental basis the genes bringing about the reactions are not in context on the genome. As another example, it is known that colanic acid gene cluster comprises of genes involved in conversion of D-Mannose to GDP-L-Fucose. Our results using keywords along with ‘gene cluster’ showed only two papers while 23 publications were obtained in all which occurred in atleast four of the six queries. Thus, utilizing keywords like ‘gene cluster’ and ‘operon’ in query strings helps to narrow down to specific hits for this reactant-product pair. Despite narrowing down the results with 6Q query structure, these results were manually verified for all pathways and their corresponding modules in order to remove false positives. For e.g., while searching for a module with initial reactant L-Xylulose and product as L-Arabinose only one Pubmed ID 19393548 (Sakakibara et al., 2009) is found where keyword ‘operon’ also appears and this publication refers to a genetic engineering experiment where enzymes capable of this conversion have been cloned into *E. coli* in an operonic arrangement. This might lead to a spurious association of keyword operon with this module and in such cases, manual removal was necessary.

Contrary to the above mentioned examples some modules do not show any hits when keywords include ‘ReactantString+ProductString+Bacteria+Gene cluster’ or ‘ReactantString+ProductString+Bacteria+operon’ but contain Pubmed matches in the other four queries. In such cases verification is required to see if consensus Pubmed Ids obtained from the remaining four queries provide information about reference organisms. The genes corresponding to the given module in these reference organisms can then be checked for juxtaposed occurrence on their respective genomes. Such searches might help in cases where literature has information about conversion of a reactant to a product but does not indicate presence of constituent genes as operons or gene clusters. For example, search of reactant Glutamate and product Citrulline did not give any hits with keywords ‘operon’ and ‘gene cluster’ while other four query strings provided one consensus pubmed ID 25492493 (Hao et al., 2015). This publication discussed about conversion of Glutamate to Citrulline in *Corynebacterium glutamicum* wild type bacteria. Analysis of the location of constituent genes on the sequenced genomes of these bacteria showed that they occurred in context on the genome thereby confirming the module. Similarly glutamate conversion to Ornithine involved two pathways out of which one utilized *Lys*W pathway and keywords provided no publication that discussed about juxtaposed occurrence of these genes. The other four queries gave 23434852 as a consensus pubmed ID corresponding to organism *Sulfolobus acidocaldarius* and it was found that genes corresponding to this module occurred in context in these genomes. Similar observations were made for D-perosamine biosynthesis from GDP-Mannose as initial substrate.

All the above mentioned examples indicate the importance of utilizing all six queries while performing the literature search. It was observed that some modules might give correct reference organisms utilizing only first four query structures (as mentioned in section “Materials and Methods”) as no publications corresponding to last two query structures are available. On the contrary, in other cases the last two queries become necessary to narrow down to more relevant reference organisms. Despite the extraction of ∼80% of modules correctly using automation methods, 20 modules had to be searched manually and added to the database due to no consensus publications referring to those modules using these six query structures. In summary, the application of 6Q method along with extensive manual curation allowed to define biologically functional modules in each of the 2745 genomes.

#### Comparison of FLIM-MAP GCMs With KEGG MODULES

Our approach utilizes three strategies (i) identifying domain constitution on the genome and assignment of gene clusters (ii) identification of functional units/modules based on this gene context information (iii) validation using the novel ‘6Q’ literature mining strategy. Although, methods like KEGG MODULE identify modules within pathways using manual curation, information about various modules is still not curated completely. For example, despite the importance of butyrate production by gut commensal bacteria in human health and the presence of well characterized metabolic pathways, the modules corresponding to butanoate metabolism pathway (map00650) are absent in the KEGG MODULE repository. Our analysis revealed presence of five modules in this KEGG pathway which were validated for their biological relevance by literature mining. Gene context based methods help to identify modules (Pyruvate -> Butyrate, 2-oxoglutarate -> Butyrate and 4-aminobutyrate -> Butyrate) which have been extensively studied in literature (Vital et al., 2014; Anand et al., 2016). In addition to absence of modules for certain pathways, KEGG module repository does not correctly identify certain biologically relevant and experimentally validated modules in bacteria. Apart from some modules being absent in the repository, few assignments of compounds to modules are also incorrect pertaining to bacterial systems. As discussed before, it has been observed that the metabolism of D-Mannose 6-phosphate to GDP-L-Fucose of the KEGG pathway-00051 (fructose mannose pathway), has been experimentally proven to exist as an evolutionarily conserved *man* gene cluster. KEGG module entry shows that D-Mannose 6-phosphate, D-Mannose-1-Phosphate and GDP-D-mannose (which constitute first three compounds of bacterial conversion of D-Mannose to GDP-Fucose) are mapped to the KEGG Module M00114 (ascorbate biosynthesis). This affiliation in KEGG refers to a plant metabolism pathway where GDP-D-Mannose is further channeled into ascorbate biosynthesis via formation of GDP-L-Galactose. Thus, module definition in KEGG completely misses out on utilization of GDP-D-Mannose in bacteria. Therefore, incomplete curation of information might lead to inaccurate definition of metabolic potential in case of bacteria.

#### ‘FLIM-MAP’ Algorithm

**FIGURE 3:**
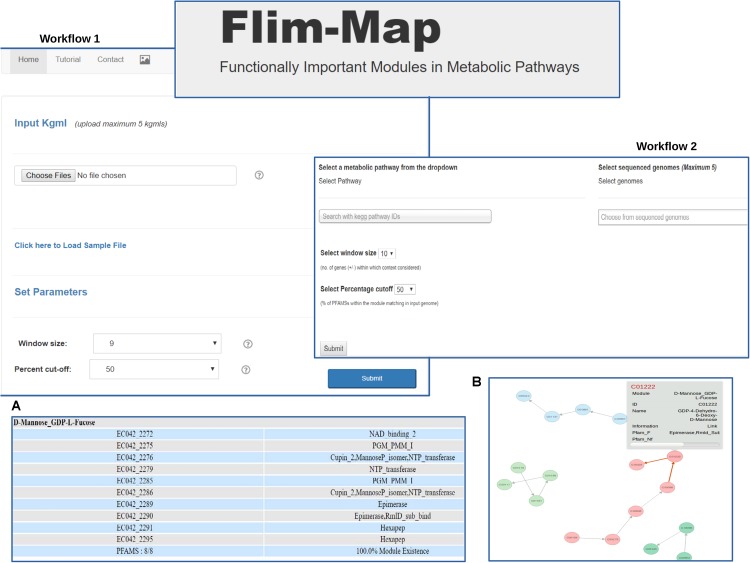
The web based tool FLIM-MAP provides the user with two workflows. User can choose to either upload pathway information files in the kgml format for bacterial genomes (Workflow I) or select the pathway of choice and organisms from a dropdown provided (Workflow II). Output can be visualized in a tabular format **(A)** and via the graphical representation of the network formed **(B)**.

In order to view and identify the extent of presence of ‘Gene Context based Modules’ (GCM’s) within a KEGG defined pathway in any prokaryotic organism; a tool called ‘FLIM-MAP’ has been developed (**Figure 3**). User is provided with the option of following two pipelines while using FLIM-MAP namely ‘Upload KGML’ and ‘Selection’ of a pathway in an organism. A KEGG ‘pathway’ is a graphical object comprising of ‘entry’ element (i.e., compounds) as its nodes and edges are formed by the ‘relation’ and ‘reaction’ elements. Such a graphical outline indicates the connection pattern between substrate and product catalyzed by a gene product (i.e., an enzyme). The former pipeline allows the user to upload an organism specific KGML file (as obtained from KEGG pathway database or user created file in similar format) for a carbohydrate or amino acid pathway listed in KEGG. User can further choose the cut-off for the percentage of total Pfams tagged to a GCM in DB2 which need to be present in order for a gene set in a query genome to be considered a valid GCM. It should be noted that for GCMs where partial matches can also be considered valid use of lower cut-offs is recommended. The interface also provides the user with an option to choose the window size which refers to the number of flanking genes to be considered while defining gene context. These two features control the stringency of the query search. A demonstrative example has been made available on the page for the user’s convenience. The latter ‘Selection’ pipeline lets the user select any one of the 2745 complete NCBI genomes for a selected carbohydrate/amino acid KEGG defined pathway (using the KEGG mapid). For a selected cut-off value and window size, all the potential modules and the extent of domain presence within the chosen window size for gene context is estimated. Results of the algorithm for both pipelines can be viewed in a network representation as well as in a tabular format and can be further downloaded as a text file. A detailed tutorial is available on the server for ease of understanding.

## Conclusion

The host–microbiome and microbiome–microbiome interactions lead to formation of an intricate ecosystem wherein resident microbes are known to influence host health and well being. This indicates the importance of understanding the ‘functional potential’ of the organisms, in terms of the metabolic pathways they possess. Despite the availability of large amount of sequence data, correct annotation of genes and their accurate pathway assignment still remains a challenge. An accurate estimation of pathway presence within an organism depends not only on the mere presence of a set of genes, but also on the contextual occurrence of the participant genes on the bacterial genome. In our study, we present a perspective for prioritizing gene context to accurately identify functional modules within bacterial genomes. Therefore, genomic context can help to differentiate the correct homolog for a pathway from its other copies on a genome lying on distant locations. We implemented the above concept in an easy to use web based framework called FLIM-MAP that can be used by biologists inexperienced in programming.

The existing gene context based methods (e.g., GeCont, MGcV, PhydBac, EFI-GNT etc.) (Ciria et al., 2004; Enault et al., 2005; Overmars et al., 2013; Gerlt, 2017) provide information about gene neighborhood of a candidate gene and also their operonic associations. These tools are based on the idea that genes of the same neighborhood are functionally associated and extend the same to search for orthologs in bacterial genomes. The pathway affiliations and hence, the biological functions performed by gene clusters are not completely curated in these repositories. Our method not only provides gene context information, but also utilizes it to define biologically relevant modules, thus providing an estimation of the functional potential in that genome. We demonstrated the utility of our tool using various case studies and expect it to be a valuable contribution in the field.

Although, our method proves to be promising in correcting metabolic pathway annotations in bacteria, certain limitations need to be noted. There are cases where multiple gene clusters might possess similar domain constitutions and enzymatic reactions but differ in the starting substrate consumed. For example, Branched-Chain α-keto Acid Dehydrogenase (BCKD), an important part of Branched Chain Amino Acid (BCAA) catabolism, forms a gene cluster containing three genes. This cluster comprises of two subunits of the branched-chain α-keto acid decarboxylase (E1), a lipoamide acyltransferase unit (E2), and a lipoamide dehydrogenase unit (E3). Although, the enzyme forms a gene cluster, similar domain constitution is observed in other enzymes like Pyruvate dehydrogenase as well as 2-oxoglutarate dehydrogenase. In such cases, multiple copies of this gene cluster can be present on a bacterial genome (e.g., *Micrococcus luteus*) and it will not be possible to identify actual BCKD gene cluster using just the gene context based methods (Surger et al., 2018). In such cases, the presence of a gene cluster comprising all constituent domains is not sufficient. The active site specificities of these clustered enzymes acting on different compounds also need to be examined in order to determine the starting substrate consumed. Also, the method is restricted to bacterial metabolic pathways for which gene cluster information is available in literature. Pathways comprising genes which do not form conserved gene clusters might not be annotated within the scope of this method (e.g., constitutively utilized pathways like Glycolysis, Glycine metabolism). It should also be noted that gene context based methods might have limited applications in eukaryotic systems where genes functional in a pathway do not show significant co-occurrence on the genome.

## Materials and Methods

**FIGURE 4:**
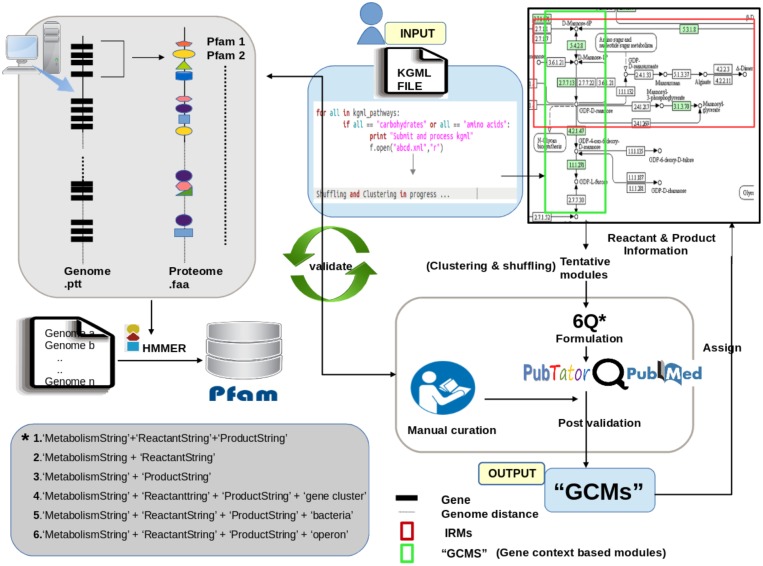
Schematic representation of the methodology used to create GCMs.

The various steps involved in functioning of FLIM-MAP are depicted in **Figure 4** and described in details below:

### HMM Based Profiling of the Bacterial Genome

The fasta sequences and ptt files corresponding to 2745 completely sequenced bacterial genomes were downloaded from NCBI^1^. The ptt files were used for including the genomic arrangement information of various genes. The domain compositions of all the genes in these bacterial genomes were obtained by Hidden Markov Model (HMM) based profiling against the Pfam database using HMMER tool (Finn et al., 2011, 2014). The annotated genes along with their domain constitutions (Pfam) were cataloged as per their locations on the genome for each organism using the NCBI ptt files as reference. This exhaustive curated catalog was utilized for gene context based clustering and shuffling (details in following sections).

### Creation of Edge Based ‘Initial Reference Modules’ (IRMs)

The edge information corresponding to each pathway in KEGG was used as a reference to manually annotate and create potential functional modules (PFMs). The reference kgml files representing the structure of metabolic pathways (carbohydrates and amino acids) were first downloaded to create an edge list depicting the connections between compounds (all reactants and products) involved in the pathway. Constitutive pathways like Glycolysis, which mostly comprise of housekeeping genes, were excluded from the analysis. Community detection algorithms were used to create edge connectivity based modules (using iGraph in R package). The formed clusters were derived based on the edge connectivity and were used as the ‘Initial Reference Modules (IRM).’

Organism specific pathway files (kgmls) for 2745 completely sequenced bacterial genomes were downloaded from KEGG^2^. A map was created using connection information between compounds within an IRM and the genes catalyzing the reaction. Gene information for a prokaryotic pathway was retrieved from the individual organism specific kgml file. Functional domains (Pfam) for its corresponding gene within a module were mapped using the HMM based profiling as mentioned above. The next task was to assign module identifier to those genes which connected two modules and were not part of any of the formed clusters. Such genes, termed as ‘linker genes,’ were reassigned to both the connecting modules which were later segregated using our algorithm (described below).

### Creation of ‘Gene Context Based Modules’ (GCMs) for Estimation of Potential Function

The created Modules were compared in pairs, extending over nC2 combinations, where *n* represented the number of IRMs in a pathway. Information on gene locations which were recorded during HMM profiling was used to calculate the average distances between the genes. Both inter-modular gene distances as well as intra modular gene distances were determined. Proximal genes were clustered together to form new intermediate modules (IM). This step was recursively performed till all the genes were segregated into the clusters which were classified on the basis of gene context. Inter cluster distances were calculated and based on minimum distance score (chosen as 10) the clusters were either merged or kept as they were, thus forming redefined modules. This methodology was executed for all the 2745 complete bacterial genomes.

Validation as well as refinement of the redefined modules was done using extensive literature mining and manual curation. Literature mining was performed using the ‘6Q Approach’ (detailed below), results of which contained the list of consensus organisms for each query sub-module within a KEGG defined pathway. Functionally relevant domains associated within a module were manually curated and analyzed across the consensus organisms obtained from the literature search. Post shuffling and refinement, a mapping between each module and the compounds as well as functionally important Pfams was created. These reformulated biologically relevant modules with specific metabolic activities are termed as ‘Gene Context based Modules’ (GCMs) (**Figure 3**).

### ‘6Q Approach’

Post identification of IMs, extensive mining of literature data became essential in order to validate the results. This was done by using an automated literature mining approach called ‘6Q strategy’ as well as manual curation. The process of automated literature mining was performed using a unique systematic keyword searching method called ‘6Q strategy.’ Each of the IMs was considered a sub-system comprising of a series of enzymatically catalyzed steps acting on a reactant consequently yielding a product. These sub-systems defined within a KEGG pathway were manually checked extensively to further refine the reactant and product information.

The 6Qs (queries) explicated as:

• ‘ReactantString’ (Query 1)
• ‘ProductString’ (Query 2)
• ‘ReactantString’ + ‘ProductString’ (Query 3)
• ‘ReactantString’ + ‘ProductString’ + ‘bacteria’ (Query 4)
• ‘ReactantString’ + ‘ProductString’ + ‘operon’ (Query 5)
• ‘ReactantString’ + ‘ProductString’ + ‘gene cluster’ (Query 6)

The ‘ReactantString’ is a manually curated string identifier for source metabolite/reactant and ‘ProductString’ is a string identifier for target metabolite/product. The ReactantString and ProductString identify the first reactant and the final product of the corresponding module, respectively. KEGG repository was further mined to obtain all synonyms for each of the compounds used as ReactantString and ProductString. All these synonyms for ‘ReactantString’ and ‘ProductString’ were used as query while performing literature search. The above mentioned queries were then used as input to search against the curated biomedical literature resources, namely Pubmed and Pubtator (Wei et al., 2013). The resultant hits so obtained contained ‘Pubmed IDs’ sorted by relevance for each query. Consensus Pubmed IDs occurring in at least 4 of the 6 query lists were filtered out. A map was created for Pubmed IDs and corresponding organisms using the Pubtator knowledgebase. This helped to obtain reference organisms for each of the IMs identified. Further, the reference organisms as well as literature information obtained from 6Q approach were utilized to check the constitution of each IM and further refine them manually in order to remove discrepancies. The Pfams tagged to each IM were also confirmed using Pfams corresponding to genes carrying out each of the steps included in an IM in identified reference organisms. Further, validations the IMs which did not yield satisfactory results after using 6Q approach were also searched in literature manually in order to include this modules which might have literature validation but do not correctly fit into the six query keywords listed above.

### Web Server Development

In order for the user to view and identify the extent of presence of GCM’s within a KEGG defined pathway, we developed the tool called ‘FLIM-MAP.’ The identified GCMs are graphically presented with distinctive colors in an interaction network. This web application was designed on the ‘Model View Controller (MVC)’ architecture. Model and Controller elements were built using MySql and PHP, Python, respectively. User interface and graphical visualization was created using HTML5, Javascript, Jquery and d3.js.

## Availability

https://web.rniapps.net/FlimMap [Freely available for academic use].

## Author Contributions

SA, BK, and SM conceived the idea. VB and AM designed the overall methodology, implemented the algorithms, and developed the web server. SA, VB, and AM performed the case studies. VB, AM, SA, BK, and SM analyzed the results and drafted the manuscript. All authors read and approved the final manuscript.

## Conflict of Interest Statement

The authors declare that the research was conducted in the absence of any commercial or financial relationships that could be construed as a potential conflict of interest.

## Footnotes

1 ftp://ftp.ncbi.nlm.nih.gov/genomes

2 http://www.genome.jp/kegg/pathway.html
