# Corrigendum: FLIM-MAP: Gene Context Based Identification of Functional Modules in Bacterial Metabolic Pathways

**DOI:** 10.3389/fmicb.2020.605419

**Published:** 2020-10-29

**Authors:** Vineet Bhatt, Anwesha Mohapatra, Swadha Anand, Bhusan K. Kuntal, Sharmila S. Mande

**Affiliations:** ^1^Bio-Sciences R&D Division, TCS Research, Tata Consultancy Services Ltd., Pune, India; ^2^Chemical Engineering and Process Development Division, CSIR-National Chemical Laboratory (NCL), Pune, India; ^3^Academy of Scientific and Innovative Research (AcSIR), Ghaziabad, India

**Keywords:** gene context, functional potential, bacteria, metabolic pathway, annotation, genome

In the published article, there were errors in the affiliation list.

Affiliation 1, “Bio-Sciences R&D Division, TCS Research, Tata Consultancy Services, Pune, India,” should appear as “Bio-Sciences R&D Division, TCS Research, Tata Consultancy Services Ltd., Pune, India”

Affiliation 2, “Academy of Scientific and Innovative Research (AcSIR), CSIR-National Chemical Laboratory, Pune, India,” should be listed as two different affiliations: “2 Chemical Engineering and Process Development Division, CSIR-National Chemical Laboratory (NCL), Pune, India” and “3 Academy of Scientific and Innovative Research (AcSIR), Ghaziabad, India.”

In the published article, there was an error regarding the affiliations of Bhusan K. Kuntal. As well as having affiliations 1 and 2, they should also be affiliated with 3.

In the published article, the Conflict of Interest statement was incorrect. It should read:

All the authors were employed by Tata Consultancy Services Ltd.

The authors apologize for these errors and state that this does not change the scientific conclusions of the article in any way. The original article has been updated.

