# Supplementary material for: FLIM-MAP: Gene Context Based Identification of Functional Modules in Bacterial Metabolic Pathways
Source: Front Microbiol. 2018 Sep 18;9:2183. doi: 10.3389/fmicb.2018.02183 (PMC6157337; doi:10.3389/fmicb.2018.02183)
Supplement: Supplementary file 2 [file Image_1.PDF]

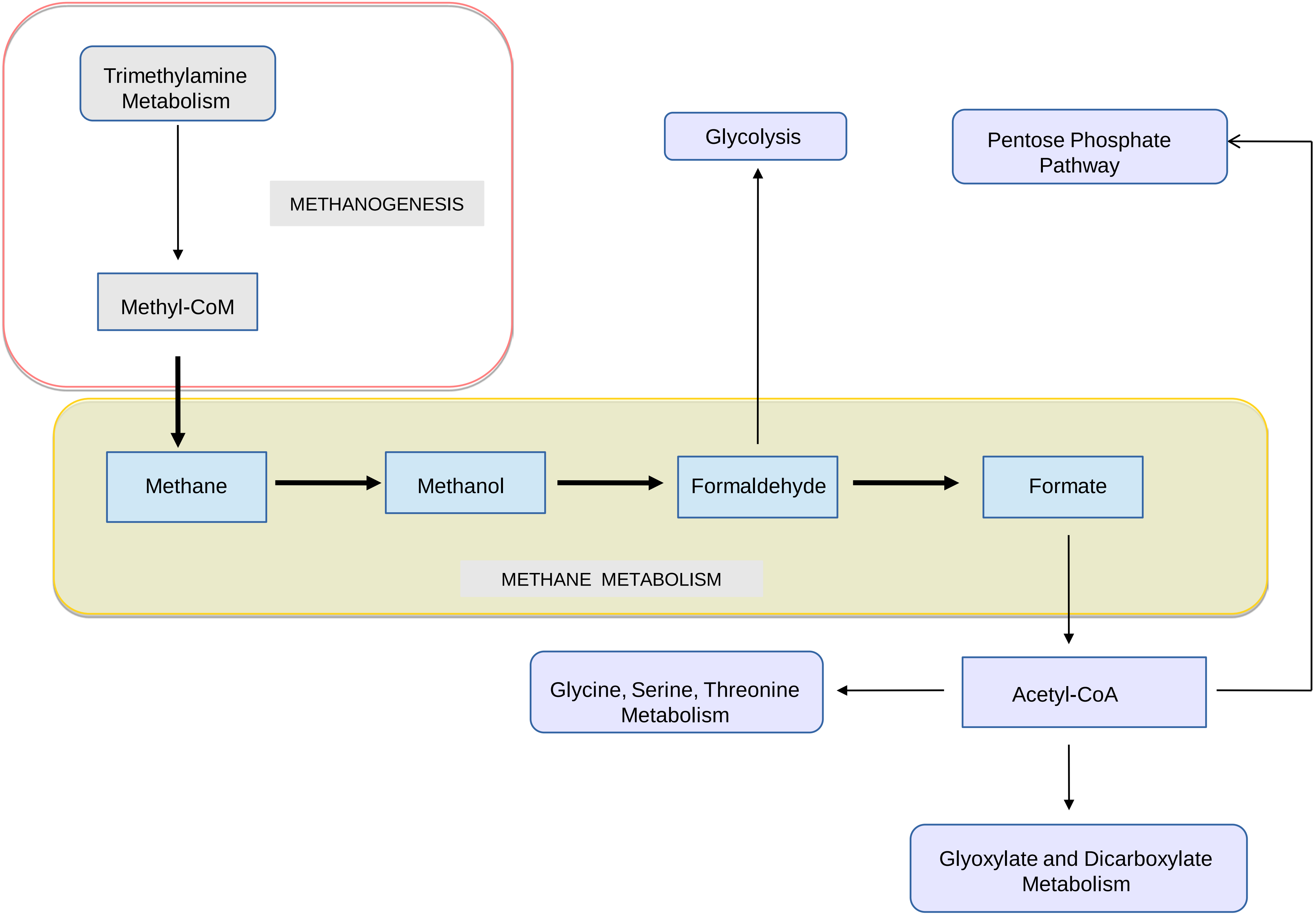

Supp. Fig. S1: Figure showing gene cluster involved in methane metabolism highlighted as a shaded box.
