# Supplementary material for: FLIM-MAP: Gene Context Based Identification of Functional Modules in Bacterial Metabolic Pathways
Source: Front Microbiol. 2018 Sep 18;9:2183. doi: 10.3389/fmicb.2018.02183 (PMC6157337; doi:10.3389/fmicb.2018.02183)
Supplement: Supplementary file 3 [file Image_2.PDF]

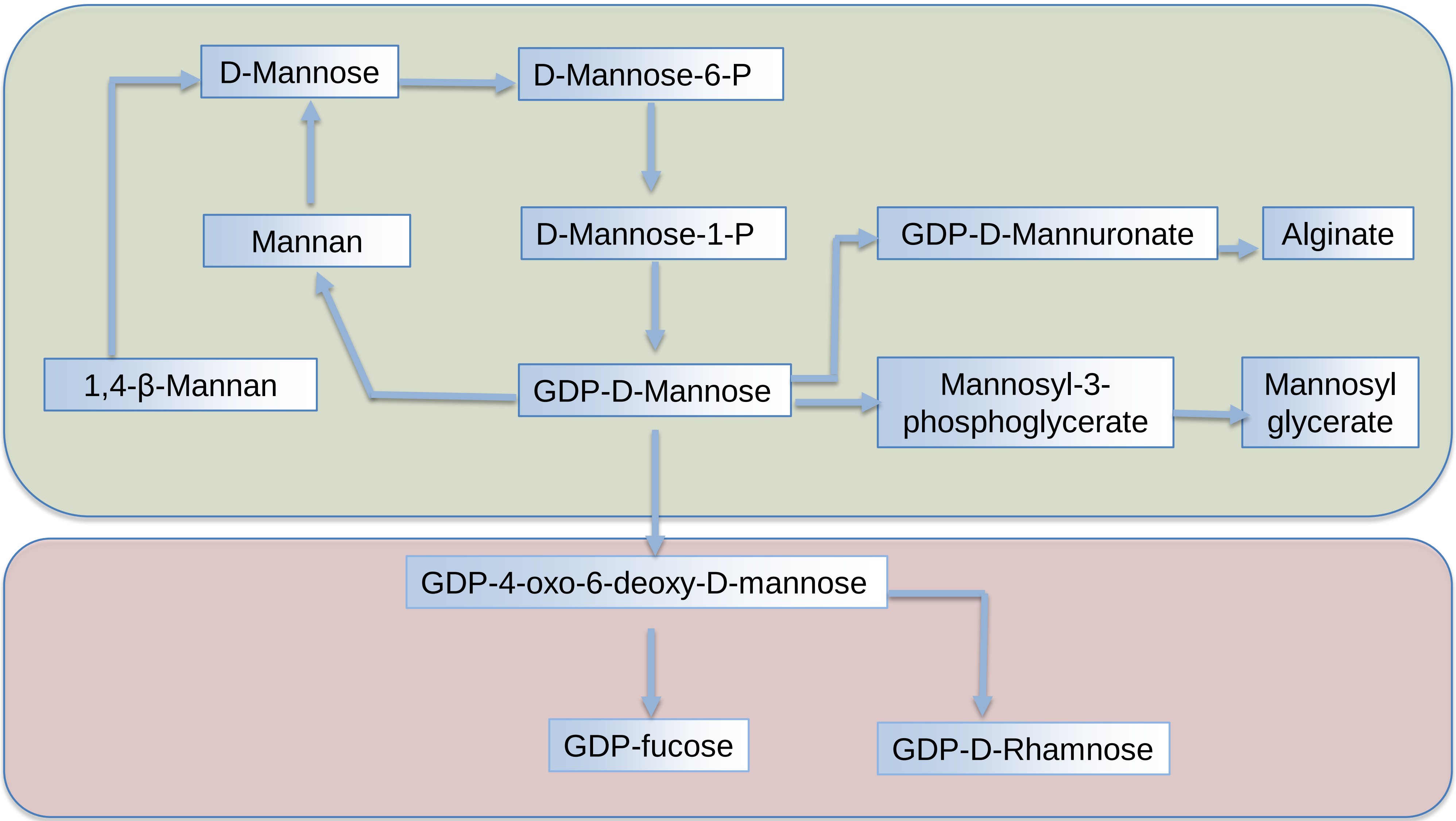

Supp. Fig. S2: The edge connectivity based modules (fast greedy) divided the pathway from D-Mannose-6-phosphate to GDP-fucose into two separate modules. Thus taking all connections of KEGG for these pathways is not biologically significant.
