# Supplementary material for: FLIM-MAP: Gene Context Based Identification of Functional Modules in Bacterial Metabolic Pathways
Source: Front Microbiol. 2018 Sep 18;9:2183. doi: 10.3389/fmicb.2018.02183 (PMC6157337; doi:10.3389/fmicb.2018.02183)
Supplement: Supplementary file 4 [file Image_3.PDF]

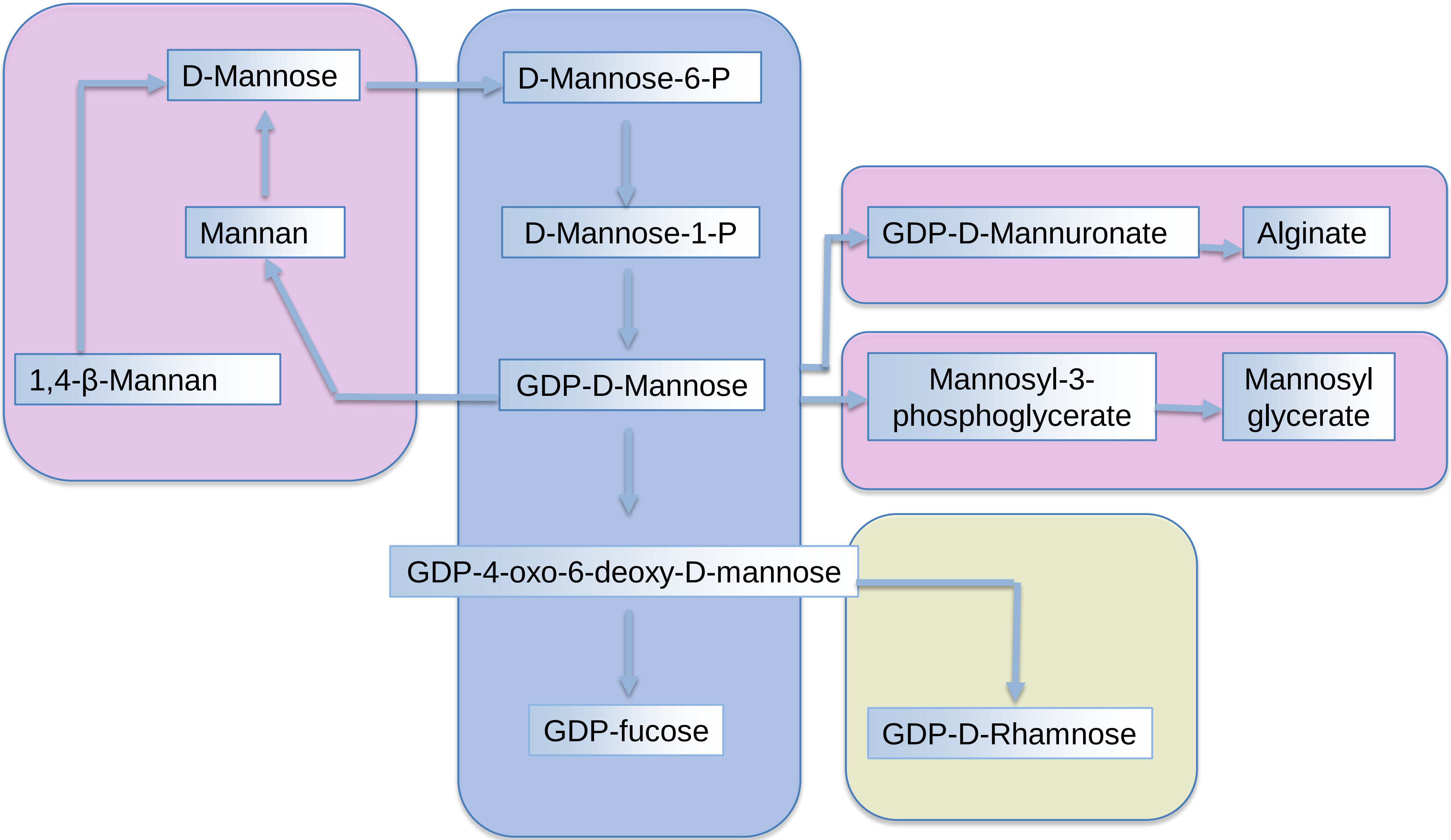

Supp. Fig. S3: The FLIM-MAP algorithm is able to identify and segregate the biologically relevant functional units in the Fructose-mannose metabolism pathway highlighted in the blue shaded box
